# Voluntary Running and Estrous Cycle Modulate ΔFOSB in the Suprachiasmatic Nucleus of the Wistar Rat

**DOI:** 10.5334/jcr.257

**Published:** 2025-05-19

**Authors:** Ayano Shiba, Marene H. Hardonk, Ewout Foppen, Tess Kool, Susanne E. la Fleur, Paul J. Lucassen, Chun-Xia Yi, Dirk Jan Stenvers, Joram D. Mul, Andries Kalsbeek

**Affiliations:** 1 Netherlands Institute for Neuroscience, Institute of the Royal Netherlands Academy of Arts and Sciences (KNAW), Meibergdreef 47, 1105BA Amsterdam, The Netherlands; 2 Amsterdam UMC, University of Amsterdam, Laboratory of Endocrinology, Department of Laboratory Medicine, Meibergdreef 9, 1105AZ, Amsterdam, The Netherlands; 3 Amsterdam Gastroenterology, Endocrinology and Metabolism (AGEM), Amsterdam, The Netherlands; 4 Brain Plasticity group, Swammerdam Institute for Life Sciences, Faculty of Science, Science Park 904, 1098XH Amsterdam, The Netherlands; 5 Centre for Urban Mental Health, University of Amsterdam, Amsterdam, The Netherlands; 6 Department of Endocrinology and Metabolism, Amsterdam UMC, University of Amsterdam, Meibergdreef 9, 1105AZ, Amsterdam, The Netherlands

**Keywords:** Voluntary wheel running, Exercise, Circadian rhythm, SCN, brain clock, Immediate early gene

## Abstract

The hypothalamic suprachiasmatic nucleus (SCN), the circadian pacemaker of the mammalian brain, integrates both environmental and endogenous information to modulate various physiological and behavioral processes. Both light and physical activity entrain SCN circadian rhythmicity, but the underlying molecular mechanisms for physical activity remain elusive.

Repetitive neuronal stimulation results in accumulation of the stable transcription factor ΔFOSB, that has been implicated in long-term brain plasticity, altered neuronal excitability, and changes in behavior. In rodents, voluntary wheel running (VWR) mimics aspects of exercise training and increases ΔFOSB in several brain regions. Whether VWR also alters ΔFOSB in the SCN is unexplored.

Here, young-adult male and female Wistar rats were housed sedentary or allowed to run for four weeks followed by quantification of ΔFOSB in the SCN. VWR lowered SCN ΔFOSB-positive cell numbers in males and females compared to sedentary housing. Total running distance did not correlate with ΔFOSB suppression. Analysis taking estrous cycle into account revealed that ΔFOSB-positive cell numbers were cyclic in sedentary females, being lowest during proestrus and highest during diestrus. Remarkably, this cyclicity was absent in runners, where ΔFOSB-positive cell numbers remained comparable to those observed during proestrus in sedentary controls. Finally, estradiol replacement following ovariectomy in sedentary females lowered SCN ΔFOSB-positive cell numbers.

Thus, VWR and estrous cycle, via, at least in part, estradiol, modulate SCN ΔFOSB. Given its role in long-term plasticity and behavioral adaptations, ΔFOSB may provide a molecular link between VWR and/or estrous cycle and the output of the SCN and its related behavioral adaptations.

**Figure:**
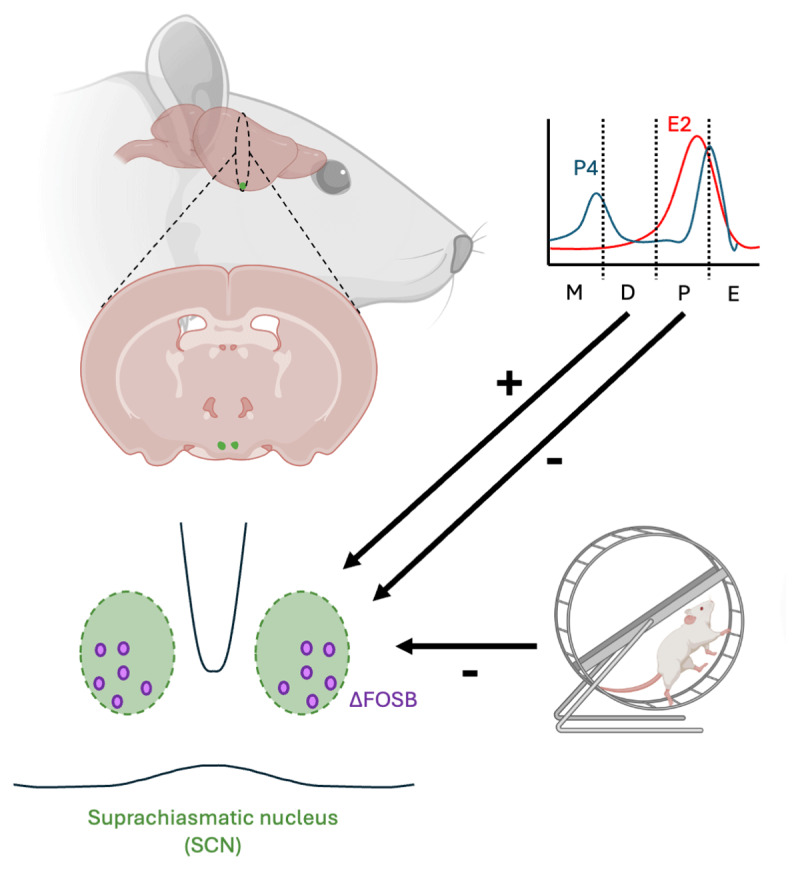


## Introduction

The suprachiasmatic nucleus of the hypothalamus (SCN) is the circadian pacemaker of the mammalian brain. It enforces daily rhythms in several physiological and behavioral processes, including those in energy homeostasis, various hormones, sleep/wake cycles and physical activity. The SCN integrates external and internal information to entrain its own circadian rhythmicity to the 24h environmental light/dark cycle. Important entrainers, or zeitgebers, of the SCN are exposure to light, consumption of calories, and physical activity [1,2].

A commonly used behavioral paradigm to monitor daily SCN output in rhythmicity is voluntary wheel running (VWR). In rodents, VWR is a self-reinforcing behavior, that can profoundly impact plasticity of the brain [3,4,5,6,7,8]. Access to a running wheel can *e.g*. affect circadian rhythmicity and output of the SCN, depending on the amount, intensity and timing of VWR [9,10,11]. Moreover, VWR, when performed during the active (*i.e*. dark) phase of rats, enhances the amplitude of SCN oscillations [12]. Next to VWR, another cyclic input to the SCN is the female hormonal cycle and locomotor activity patterns are e.g. influenced also by the estrous cycle. Although several studies have investigated the impact of VWR on the central (SCN) and peripheral clocks [12,13,14], the underlying molecular mechanisms as to how VWR can impact the SCN, and whether this occurs in a sex-dependent manner, remain poorly understood.

ΔFOSB is a highly stable transcription factor that mediates activity-dependent gene expression and is involved in long-lasting molecular and structural adaptations in neuronal circuits involved in reward processing and addiction, memory, and stress resilience [15,16,17,18,19,20]. ΔFOSB is a family member of the FOS family of immediate early gene activator protein-1 (AP-1) transcription factors and is generated via alternative splicing of the parent *Fosb* mRNA. ΔFOSB regulates gene expression via self-assemblies and via the formation of heterodimers with JUN proteins, such as JUND, to form AP-1 transcription complexes [16,21,22,23]. Due to several structural modifications, ΔFOSB has a remarkable long half-life compared to other FOS-related transcription factors and can persist for days or weeks in neurons where it accumulates when they are stimulated repetitively [24,25,26,27,28,29].

Given its role in reward, memory, and stress, studies that investigated ΔFOSB have so far focused on striatal, cortical or hippocampal brain regions [15,30,31,32]. However, FOSB and ΔFOSB are also expressed in the hypothalamus, where high levels are observed in the SCN [33,34,35,36,37]. Although these studies have revealed differential regulation of FOS family proteins in rodent SCN in response to light, it is currently unclear if ΔFOSB is involved in the effects of VWR on the circadian timing system. Due to its high stability, ΔFOSB can regulate gene expression and may thus modulate physiological processes and neuronal function for an extended timeframe. Whether sex differences exist was unclear and we therefore here investigated if four weeks of voluntary running would modulate SCN ΔFOSB levels, both in male and female Wistar rats.

## Materials and methods

### Ethics approval statement

All experimental procedures were performed in accordance with the European guidelines for laboratory animals (EU directive 2010\63\EU) and approved by the Dutch Central Committee for Animal Experiments (CCD; AVD11800202316744, AVD80100202216157 and AVD8010020172424) and the Agency for Animal Welfare (IvD) of the Netherlands Institute for Neuroscience (NIN; Royal Dutch Academy of Sciences). To minimize general distress, rats were housed in cages with corncob bedding and cage enrichment [an aspen wood gnawing stick (Technilab-BMI) and a PVC shelter (Bio Services)]. Furthermore, rats were housed in open cages and in the same experimental room, which enables them to smell and hear each other. This was done in an attempt to counterbalance the solitary housing during the experiment, which was necessary to adequately measure individual running distance and caloric intake.

### Animal cohorts

#### Housing conditions

A total of 28 male and 76 female Wistar WU rats [Crl:WI(WU), Charles River, Germany] were used for these experiments. All rats were housed in a temperature- (21–23°C), humidity- (40–60%) and light-controlled experimental room [12:12h light/dark cycle; 280 (±80) Lux (lights on), <5 Lux (lights off); with lights on at Zeitgeber time 0 (ZT0) and lights off at ZT12] in the NIN animal facility. A sound system continuously played soft radio music during the experiments to provide background noise. To recover from transport stress and habituate to the animal facility, rats were group-housed (4/cage) in a polycarbonate type 4 cage [530(l) × 330(w) × 200(h) mm; 1815 cm2; Plexx] for one week upon arrival at the NIN. During acclimatization and experiments, rats had *ad libitum* access to a bottle of tap water and an irradiated nutritionally complete high-carbohydrate diet (Teklad global diet 2918, 24% kcal from protein, 58% kcal from carbohydrate, and 18% kcal from fat, 3.1kcal/g, Envigo).

#### Primary male and female running cohorts for SCN ΔFOSB analysis

After 6–8 days of acclimatization to the NIN animal facility, male and female rats (weighing 255–270 grams and 170–200 grams, respectively, upon arrival; approximately 8–9 weeks old) were individually housed and randomly assigned to one of two experimental groups: sedentary controls (male, n = 14; female, n = 12) were housed without access to a running wheel in a polycarbonate type 3H cage [375 (l) × 215 (w) × 180 (h) mm)], whereas runners (male, n = 14; female, n = 12) were housed in custom-made cages [522 (w) × 582 (l) × 412 (h) mm] with free access to a stainless-steel vertical wheel (width: 11 cm, diameter: 35.6 cm, 1.1m/revolution; model 80850MS, Campden Instruments) for 29 days. Wheel revolutions were continuously registered every second using in-house developed software. The experimental groups were body weight-matched at the start of the experiment and body weight was measured every seven days. Caloric and water intake was measured every 2–3 days. Twenty-four hours prior to the end of the experiment, running wheels were blocked for runners to make sure any residual wheel running-induced full-length FOSB protein had been degraded at the time of sacrifice, and all remaining immunoreactivity primarily reflects ΔFOSB [38]. This procedure was necessary as the rabbit monoclonal antiserum that was used for the detection of the ΔFOSB signal was raised against the N-terminal region of FOSB, thereby recognizing both FOSB and ΔFOSB (5G4; #2251, Cell Signaling Technology). For female rats, a vaginal smear was taken before perfusion to determine estrous stage (*i.e*. proestrus, estrus, metestrus, and diestrus) based on the ratio of nucleated epithelial cells, cornified cells, and leukocytes present in vaginal smears. After 29 days of running, rats were deeply anesthetized between ZT5 and ZT7, *i.e*. in the middle of their inactive phase, with an overdose of sodium-pentobarbital and transcardially perfused with ice-cold 0.9% saline followed by ice-cold 4% paraformaldehyde in 0.1M phosphate-buffered saline (PBS; pH 7.4). Brains were removed and stored in 4% paraformaldehyde in PBS at 4°C for at least 24 hrs. Thereafter, for cryoprotection, brains were transferred to 30% sucrose in 1X tris-buffered saline (TBS; 50 mM Tris–Cl, 150 mM NaCl; pH 7.6] with 0.05% sodium azide and stored at 4°C until further processing. Dehydration was considered complete when the brains had fully sunk to the bottom of the container, which required at least 24 hours. Coronal brain sections (35 μm) were cut on a cryostat and stored in cryoprotectant medium (30%v/v glycerol, 30%v/v glyceraldehyde and 40%v/v 1X TBS) at –20°C. Coronal brain slices from Bregma –0.48 mm to –0.96 mm were then used for quantification of ΔFOSB levels in the (rostral to caudal) SCN (Supplemental Fig. 1).

#### Female running cohort for verification of SCN ΔFOSB analysis

Data from a second and independent female running cohort (experiment performed in a different room at the NIN with slightly smaller wheels) was used to confirm quantification of SCN ΔFOSB levels after voluntary running. After seven days of acclimatization to the NIN animal facility, female rats (weighing 170–200 grams upon arrival; approximately 8–9 weeks old) were individually housed without access to a running wheel (*i.e*. sedentary; n = 11) in a polycarbonate type 3H cage [375 (l) × 215 (w) × 180 (h) mm)] or housed with free access to a stainless steel vertical running wheel (*i.e*. runners: female: n = 11; width: 10 cm, diameter: 34 cm, 1.068m/revolution) in a custom-made cage (422 (l) × 422 (w) × 475 (h) mm) for 28 days. Wheel revolutions were continuously registered using an in-house developed Cage Registration Program (Dep. Biomedical Engineering, UMC Utrecht, The Netherlands). Two male transgenic ChAT::*Cre* Long-Evans rats were housed in the same room from the start of the experiment in an attempt to synchronize female estrous cycles. Twenty-four hours prior to the end of the experiment, running wheels were blocked for runners to make sure any residual wheel running-induced full-length FOSB protein had been degraded at the time of sacrifice, and all remaining immunoreactivity primarily reflects ΔFOSB [38]. Rats were killed by perfusion, brains were removed and processed in the same manner as for the primary running cohorts, and a vaginal smear was taken to determine estrous stage.

#### Female verification cohort for ΔFOSB analysis during estrous cycle

A third and independent female sedentary cohort was used to further confirm quantification of SCN ΔFOSB levels during the estrous cycle. After five days of acclimatization to the NIN animal facility, female rats (n = 12; weighing 195–225 grams upon arrival; approximately 10–11 weeks old) were pair-housed and a vaginal smear was taken every day at ZT2 to determine estrous stage. After completing 2–3 estrous cycles, rats were killed in the proestrus or diestrus stage by perfusion that same day between ZT5 and ZT7, and brains were removed and processed similar to the primary running cohorts. Data from two rats (one in proestrus stage and one in diestrus stage) had to be excluded from analysis due to missing brain sections.

#### Female cohort for SCN ΔFOSB analysis following ovariectomy and estradiol replacement

A fourth female cohort was used to determine if estradiol replacement in ovariectomized female rats modulates SCN ΔFOSB. After 5–7 days of acclimatization to the NIN animal facility, 16 pair-housed female rats (weighing 170–200 grams upon arrival; 8–9 weeks old) were anesthetized with inhalation isoflurane (3–5% induction, followed by 1.5–2,5% maintenance) and ovaries were removed through two 1.5-cm lateral dorsal incisions in the retroperitoneal region. For pain relief, rats were injected with Meloxicam (1 mg/kg; Metacam, Hyperdrug C.O., United Kingdom) once before and once immediately after the ovariectomy surgery. In addition, Rimadyl (Carprofen, 0.6–1.5 mg/rat/day; Zoetis, the Netherlands) was added to the drinking water starting during the lights-on phase of the surgery day and provided for 72h. Subsequently, the drinking water was replaced with new drinking water containing either vehicle (final concentration: 0.031% ethanol; n = 8) or estradiol (875μg/L 17β-estradiol, #E8875, Sigma Aldrich; dissolved in 0.031% ethanol; n = 8) [39,40]. Rats were pair-housed within treatment condition (vehicle or estradiol). After 23 days of estradiol replacement via the drinking water, rats were killed by perfusion between ZT5 and ZT7, brains were removed and processed in the same manner as for the primary running cohorts, and a vaginal smear was taken to determine estrous stage. In addition, uteri were isolated and weighed to determine the effects of estradiol replacement on uterus size. Data from one vehicle-treated animal had to be excluded from analysis due to missing brain sections.

### Immunocytochemistry to determine the estrous stage

Vaginal epithelial cells were collected with sterile plastic swabs (#861.562.010, Sarstedt BV), placed on glass slides (#631-0457, VWR), and dried overnight at room temperature, or at 55°C for 30 min when determination of estrous stage was done right before killing of the rats. After drying, smears were stained with Giemsa solution (#48900-100ML-F, Merck) for 10 min, washed 3x with 1X TBS, dried overnight at room temperature, or at 55°C for 30 min when determination of estrous stage was done right before killing of the rats, and estrous stage was determined using a microscope (DM2000, Leica).

### Single-labeling immunohistochemistry

ΔFOSB immunoreactivity was detected using a rabbit monoclonal antiserum raised against the N-terminal region of FOSB that recognizes both FOSB and ΔFOSB, as mentioned above (5G4; #2251, Cell Signaling Technology). ΔFOSB-like staining was revealed by use of the avidin-biotin peroxidase method. Coronal brain slice sections were pre-mounted on the Superfrost ++ glass slides (Menzel), washed 3x in 1X TBS before antigen retrieval by incubation for 1.5h with Tris-citrate buffer (pH 8.0) at 95°C. Sections were then washed 3x in 1X TBS, and blocked for 45 min in 3% skim milk (Campina) in 1X TBS. Thereafter, sections were incubated for 1 hr in a humidified chamber at room temperature and then overnight at 4°C with Supermix (0.25% w/v gelatin, 0.5% v/v Triton X-100, in 1x TBS, pH 7.6) containing anti-FosB(N-terminus) antibody (1:1000; 5G4; #2251, Cell Signaling Technology). Sections were washed with TBS and incubated for 1 hr at room temperature with 10 mM sodium phosphate, pH 7.8, 0.15 M NaCl, 0.08% sodium azide, 3 mg/ml bovine serum albumin containing biotinylated goat anti-rabbit IgG (1:400; H+L; BA9200; Vector Laboratories, Burlingham) diluted in Supermix. Sections were washed with TBS and incubated for 1 hr at room temperature with avidin-biotin complex (1:800; Vectastain Elite ABC HRP Kit, Vector Laboratories) diluted in Supermix. Peroxidase activity was visualized by reaction with 0.05M TB (pH 7.6) containing 0.05% w/v diaminobenzidine (Vector Laboratories), 0.23% w/v nickelammoniumsulphate (Merck) and 0.01% w/v H_2_O_2_ (Merck). The reaction was stopped by dilution in tap water. Sections underwent dehydration with increasing concentrations of ethanol and 100% Xylene, and coverslipped with Entellan (Merck).

Sections were scanned using a Zeiss Axio Scan.Z1 slide scanner (Carl Zeiss AG, Oberkochen, Germany). Bregma of brain slices was determined based on the optic nerve lengths and the morphological features of landmarks and other brain regions in each section. Per rat, five equally spaced SCN sections (Bregma –0.48, –0.60, –0.72, –0.84, and –0.96) were used to quantify the numbers of ΔFOSB-immunoreactive nuclei within the SCN using QuPath Cell Detection [41]. The experimenter (AS) was unaware of the treatment information to avoid bias during analysis.

### Double-labeling immunohistochemistry

To link ΔFOSB expression to specific SCN cell types, immunofluorescence was used to double-label for ΔFOSB [1:100; a custom-made Alexa Fluor 488 pre-conjugated FOSB (5G4) rabbit monoclonal antibody, Cell Signaling Technology] and for vasoactive intestinal peptide (VIP; 1:500; Viper, #RRID: AB_2513212 rabbit polyclonal antibody developed in-house at the NIN) or arginine vasopressin (AVP; 1:500; Truus 86, #RRID: AB_2313977, rabbit polyclonal antibody developed in-house at the NIN). Sections were pre-mounted on the Superfrost ++ glass slides (Menzel), washed 3x in 1X TBS before antigen retrieval by incubation for 1.5 h with Tris-citrate buffer (pH 8.0) at 95°C. Sections were then washed 3x in 1X TBS, and blocked for 1hr in 10% normal donkey serum in PBS. Sections were incubated for 1 hr in a humidified chamber at room temperature and then overnight at 4°C with supermix (0.25% w/v gelatin, 0.5% v/v Triton X-100, in 1X TBS, pH 7.6) containing the primary antibodies. Sections were rinsed with 1X TBS, and incubated for 1hr at room temperature with 5 mM sodium azide and 1X PBS (pH 7.5) containing Alexa Fluor 594 goat anti-rabbit IgG (1:400, Invitrogen; for VIP and AVP) diluted in supermix. After counterstaining with 4,6-diamidino-2-phenylindole (DAPI, 1:2000, Thermo Fisher), slides were coverslipped with mounting medium (Vectashield Vibrance, Vector Laboratories). Photomicrographs for localization of protein expression were obtained using a confocal microscope (Leica TCS SP5 ll) equipped with an inverted DMi6000 with a 40x objective (HC PL APO CS2 40x1.4 oil).

### Statistics

Data are presented as mean ± SEM. Two-group comparisons were performed by two-tailed Student’s t-test. Assessment of effects in experiments involving several conditions was performed using two-way analysis of variance (ANOVA), with repeated measures where applicable, followed, when appropriate, by Tukey HSD or Sidak’s post hoc tests to adjust for multiple comparisons. A *P* value <0.05 was considered significant. See figure legends for statistical details of individual experiments, including statistical tests used, *t, P*, F-values, and number of subjects or samples tested. GraphPad Prism v10 was used to generate graphs and perform statistical analysis.

## Results

### Voluntary running behavior and physiological effects in male and female Wistar rats

**Figure 1:**
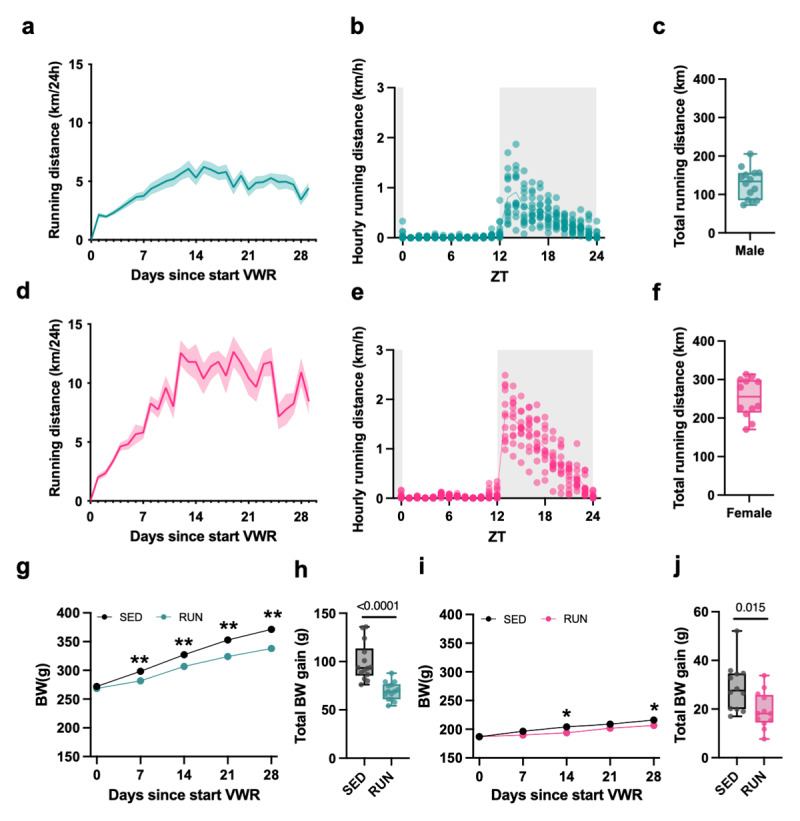
**Voluntary running behavior and impact on body weight**. **(a)** Daily running distance, **(b)** daily running patterns and **(c)** total running distance of male runners during 29 days of running. **(d)** Daily running distance, **(e)** daily running patterns and **(f)** total running distance of female runners during 29 days of running. **(g)** Body weight (BW) evolution (*time* × *housing* interaction, F_(4, 104)_ = 20.89, *P* < 0.0001; Tukey post hoc: *P < 0.05, **P < 0.01, SED *versus* RUN) and **(h)** total BW gain (t = 5.158, df = 26, *P* < 0.0001 versus SED) of male runners (RUN) and sedentary (SED) controls. **(i)** BW evolution (*time* × *housing* interaction, F_(4, 88)_ = 5.14, *P* = 0.0009; Sidak post hoc: *P < 0.05, SED *versus* RUN) and **(j)** total body weight gain (t = 2.646, df = 22, *P* = 0.0147 versus SED) of female runners (RUN) and sedentary (SED) controls. Data are presented as the mean ± S.E.M or as box plots indicating the median (line), the interquartile range, and the minimum to maximum values of the data distribution, with dots representing individual rats. a,b,c,g,h: *n* = 14/group; d,e,f,i,j: *n* = 12/group.

During the first two weeks of running, both male and female runners showed a steady increase (*i.e*. the acquisition phase) followed by a stabilization of daily running distances around 5 km/day for the male and 11 km/day for the female runners (*i.e*. the maintenance phase; Figure 1a,d). In line with the nocturnal nature of rats, most of the running was performed during the dark phase (Figure 1b,e). After 29 days of running, total running distances were around 139 ± 67 km for the male runners and around 242 ± 72 km for the female runners (Figure 1c,f). Both sedentary and running male and female rats gained body weight (Figure 1g–j), but both running groups less so compared to their respective sedentary controls (Figure 1g–j).

### Voluntary running modulates SCN ΔFOSB in male and female Wistar rats

**Figure 2:**
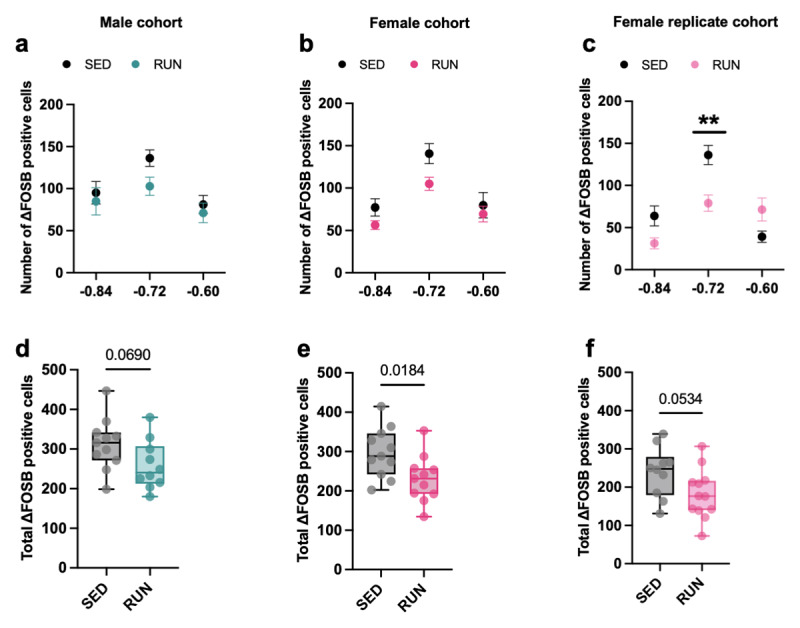
**Long-term voluntary running lowers SCN ΔFOSB**. **(a–c)** Quantification of ΔFOSB-positive cell numbers in the SCN (between bregma –0.6 mm and –0.84 mm) of **(a)** male runners (RUN) and sedentary (SED) controls (running for 29 days; *bregma* × *housing* interaction, F_(2, 38)_ = 0.5658, *P* = 0.5726; main effect of *bregma*, F_(1.65, 31.34)_ = 6.093, *P* = 0.0086; main effect of *housing*, F_(1, 19)_ = 3.715, *P* = 0.069), **(b)** female runners (RUN) and sedentary (SED) controls (*bregma* × *housing* interaction, F_(2, 40)_ = 0.7783, *P* = 0.4660; main effect of *bregma*, F_(1.256, 25.11)_ = 17.55, *P* < 0.0001; main effect of *housing*, F_(1, 20)_ = 6.586, *P* = 0.0184), and in the SCN of **(c)** an independent replication cohort of female runners (RUN) and sedentary (SED) controls (running for 28 days; *bregma* × *housing* interaction, F_(2, 40)_ = 10.83, *P* = 0.0002, Sidak’s post hoc: **P = 0.0035, SED^–0.72^ *versus* RUN^–0.72^; main effect of *bregma*, F_(1.912, 38.24)_ = 21.57, *P* < 0.0001; main effect of *housing*, F_(1, 20)_ = 4.214, *P* = 0.0534). **(d–e)** Total average number of ΔFOSB-positive cells in the SCN (between bregma –0.6 mm and –0.84 mm) of **(d)** male runners (RUN) and sedentary (SED) controls (t = 1.927, df = 19, *P* = 0.069 versus SED), **(e)** female runners (RUN) and sedentary (SED) controls (t = 2.566, df = 20, *P* = 0.0184 versus SED), and in the SCN of **(f)** an independent replication cohort of female runners (RUN) and sedentary (SED) controls (t = 2.053, df = 20, *P* = 0.0534 versus SED). Data are presented as the mean ± S.E.M **(a–c)** or as box plots indicating the median (line), the interquartile range, and the minimum to maximum values of the data, with dots representing individual rats **(d–f)**. a,d: *n* = 10–11/group; b,e: *n* = 10–12/group, c,f: *n* = 11/group.

We next quantified ΔFOSB expression in the SCN of male and female runners and sedentary controls after 29 days of voluntary running. Brain slices between bregma –0.96 mm and –0.48 mm were used to quantify the numbers of cells expressing ΔFOSB protein in the (rostral to caudal) SCN (Supplemental Figs. 1a,b). In both male and female rats, the number of SCN ΔFOSB-positive cells was lowest at the rostral and caudal SCN tails and was highest around bregma –0.72 mm (Figure 2a–c; Supplemental Figs. 1a,b).

Voluntary running for 29 days resulted in a trend for lower ΔFOSB-positive cell numbers in the SCN of male runners compared to male sedentary controls (*P* = 0.069; Figure 2a,d). In females, voluntary running for 29 days did significantly lower SCN ΔFOSB-positive cell numbers in runners compared to sedentary controls (*P* = 0.0184; Figure 2b,e).

We then used data from an independent, yet comparable, cohort of female runners and sedentary controls to replicate and confirm these observations. In this replication cohort, voluntary running for 28 days resulted in a trend for lower ΔFOSB-positive cell numbers in the SCN of runners compared to sedentary controls (*P* = 0.053; Figure 2c,f). Finally, we assessed if ΔFOSB-positive cell numbers in the SCN were related to total running distances, but we observed no significant correlations for the primary male nor the female cohort (Supplemental Figs. 2a,b). Thus, long-term voluntary running suppresses the number of ΔFOSB-positive cells in the SCN of both male and female rats, an effect that was more prominent in females than in males and was independent of total distance ran.

### SCN ΔFOSB correlates with estrous cycle in sedentary female Wistar rats

**Figure 3:**
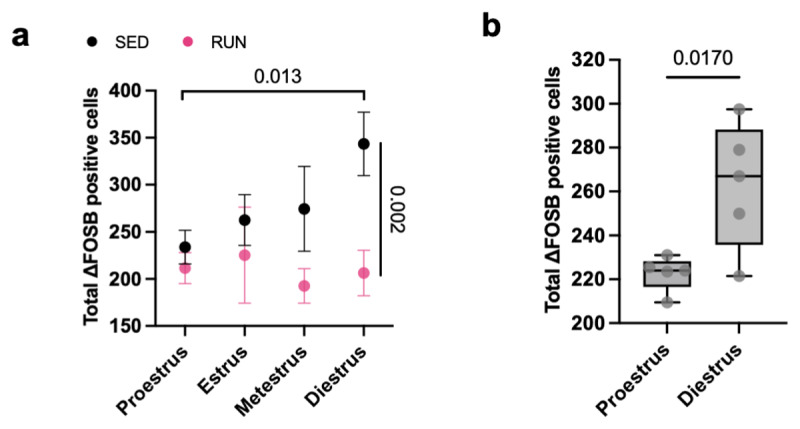
**Estrous stages modulate SCN ΔFOSB**. **(a)** Quantification of ΔFOSB-positive cell numbers in the SCN (between bregma –0.6 mm and –0.84 mm) of female runners (RUN) and sedentary (SED) controls divided by estrous stage during collection of brain tissue (main effect of *housing*, F_(1, 17)_ = 9.213, *P* = 0.0075; Tukey post hoc: *P* = 0.002, SED^Diestrus^ *versus* RUN^Diestrus^; *P* = 0.013, SED^diestrus^ *versus* SED^proestrus^). **(b)** Quantification of ΔFOSB-positive cell numbers in the SCN (between bregma –0.6 mm and –0.84 mm) of an independent replication cohort of female sedentary controls killed at proestrus or diestrus after completing at least two estrous cycles (t = 3.001, df = 8, *P* = 0.0170 *versus* diestrus). Data are presented as the mean ± S.E.M (a) or as box plots indicating the median (line), the interquartile range, and the minimum to maximum values of the data, with dots representing individual rats (b). (a) proestrus: *n* = 9–10/group; estrus: *n* = 4/group; metestrus: *n* = 3–5/group; diestrus: *n* = 4–5/group; (b) proestrus: *n* = 5; diestrus: *n* = 5.

Female locomotor activity patterns are not only strongly influenced by the circadian clock, but also by the estrous cycle. To explore whether SCN ΔFOSB levels fluctuate with the estrous cycle, data from female sedentary controls of the primary running cohort and the female running replication cohort were stratified with respect to estrous stage (*i.e*. proestrus, estrus, metestrus, and diestrus) at time of death (Supplemental Fig. 3a). Analysis of ΔFOSB-positive cells in the SCN of sedentary female controls revealed a cyclic pattern, being lowest in the proestrus phase and highest in the diestrus phase, whereas this cyclicity was absent in female runners (Figure 3a). Thus the general decrease in SCN ΔFOSB in female runners compared to sedentary controls (Figure 2b,c,e,f) appears to be primarily driven by a difference during the diestrus phase (Figure 3a). We then used data from a third cohort of only female sedentary controls to replicate and confirm these observations. The estrous cycle of these female sedentary rats was monitored daily (Supplemental Fig. 3b), and rats were killed after 2–3 complete cycles when being either in their proestrus or diestrus phase. Analysis of ΔFOSB- positive cells in the SCN in this third cohort of only female sedentary controls again revealed a cyclic pattern, being lowest in the proestrus phase and highest in the diestrus phase (Figure 3b). Thus, SCN ΔFOSB levels correlate with the estrous cycle in sedentary female Wistar rats, and this cyclicity was severely blunted in female runners.

### Estradiol replacement following ovariectomy modulates SCN ΔFOSB in sedentary female Wistar rats

**Figure 4:**
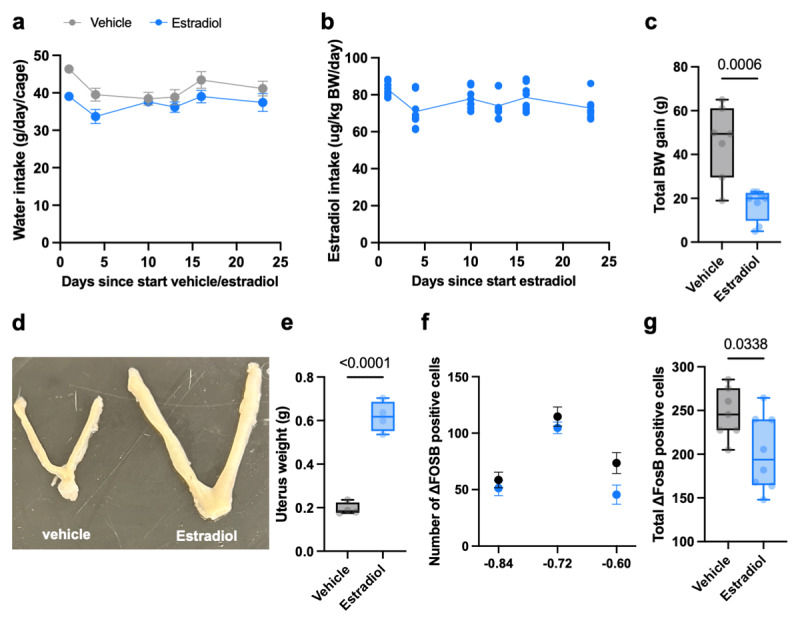
**Estradiol replacement following ovariectomy lowers SCN ΔFOSB**. **(a)** Average fluid intake (per cage) of water containing vehicle or estradiol during the 23 days of estradiol replacement. **(b)** Estradiol intake (per animal), normalized for body weight, calculated based on water intake. **(c)** Total body weight gain after 23 days of vehicle- or estradiol treatment. **(d,e)** Representative uteri (d; left, vehicle; right, estradiol) and uterus weight comparison **(e)** of vehicle- or estradiol-treated ovariectomized rats. **(f)** Quantification of ΔFOSB-positive cell numbers in the SCN (between bregma –0.60 mm and –0.84 mm) of ovariectomized rats with vehicle or estradiol (E2) replacement (*bregma* × *E2 treatment* interaction, F_(2, 26)_ = 0.9877, *P* = 0.9877; main effect of *bregma*, F_(1.533,19.93)_ = 39.71, *P* < 0.0001; main effect of *E2 treatment*, F_(1,13)_ = 5.629, *P* = 0.0338). **(g)** Total average number of ΔFOSB-positive cells in the SCN (between bregma –0.60 mm and –0.84 mm) following vehicle- or estradiol treatment (t = 2.373, df = 13, *P* = 0.0338). Data are presented as the mean ± S.E.M (d,f) or as box plots indicating the median (line), the interquartile range, and the minimum to maximum values of the data, with dots representing individual rats (b,e,g). (e) vehicle: n = 4; estradiol: n = 4, (a–c,f–g) vehicle: *n* = 7; estradiol: *n* = 8.

Circulating estrogenic hormones, including estradiol, rhythmically fluctuate over the reproductive cycle in mammalian females. Having established a correlation between SCN ΔFOSB and estrous cycle in the sedentary condition, we next tested if replacing estradiol in an ovariectomy background directly modulates SCN ΔFOSB. To do this, sedentary pair-housed female rats were ovariectomized and treated with vehicle or estradiol for 23 days via the drinking water. Daily water intake (measured per cage) indicated that estradiol consumption fluctuated within the range of 60–90 μg/kg body weight/day/animal throughout the experiment (Figure 4a,b). Estradiol replacement via the drinking water following ovariectomy was successful, as body weight gain was significantly lower in estradiol-treated rats compared to vehicle-treated controls (Figure 4c). Secondly, estradiol replacement following ovariectomy increased uterus weight compared to vehicle treatment (Figure 4d,e). Thirdly, vaginal smears taken at the end of the experiment confirmed that vehicle-treated females displayed “diestrus-stage like” characteristics, whereas estradiol-treated females displayed “late-proestrus-stage like” characteristics (data not shown). Finally, estradiol replacement following ovariectomy lowered SCN ΔFOSB-positive cell numbers compared to vehicle treatment (Figure 4f,g). Thus, estradiol can directly modulate SCN ΔFOSB in ovariectomized sedentary female Wistar rats.

### SCN ΔFOSB colocalizes with VIP, but not with AVP

**Figure 5:**
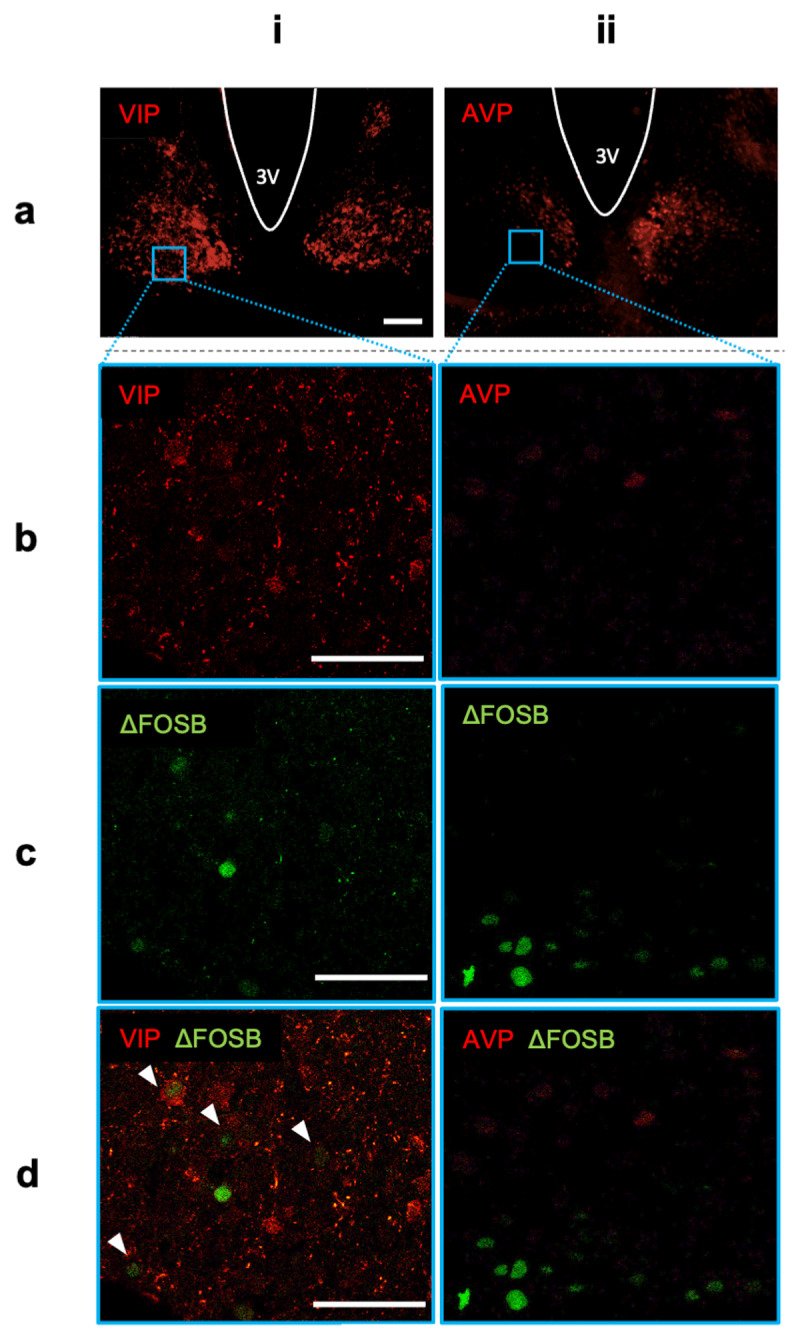
**Co-expression of ΔFOSB with VIP, but not AVP, in the SCN**. **(a**_i–ii)_ Examples of (a_i_) VIP expression and (a_ii_) AVP expression in the SCN (bregma –0.72 mm) of a female runner. **(b**_i–ii)_ Insets, shown as blue squares in a_i–ii_, at 10x magnification for (b_i_) VIP and (b_ii_) AVP. **(c**_i–ii)_ ΔFOSB expression in insets, shown as blue squares in a_i–ii_. **(d**_i–ii)_ Co-expression of (d_i_) ΔFOSB and VIP and (d_ii_) ΔFOSB and AVP in insets, shown as blue squares in a_i–ii_. White arrowheads indicate co-expression of ΔFOSB and VIP. a_i–ii_: scale bar = 100 μm; b_i–ii_, c_i–ii_, d_i–ii_: scale bar = 50 μm.

The SCN can be subdivided in two main subregions, a ventral core that contains the majority of VIP neurons, and a dorsal shell that contains the majority of AVP neurons [42]. SCN ΔFOSB is predominantly expressed in neurons in the lateral part of the ventral core, but not in the dorsal shell (Supplemental Figs. 1a,b). We next used fluorescent double-labeling immunohistochemistry to determine if neuronal VIP and AVP populations in the SCN co-express ΔFOSB. In sedentary females, we observed several examples of SCN cells with co-expression of ΔFOSB and VIP, especially in the most lateral part of the SCN (Figure 5ai–di). Such co-expression was not observed for ΔFOSB and AVP in *e.g*. the dorsal SCN (Figure 5a_ii_–d_ii_). Thus, ΔFOSB is predominantly expressed in the ventral core, but not dorsal shell, region and is co-expressed by VIP, but not AVP, cells.

## Discussion

Physical exercise has well-documented effects on circadian rhythmicity, but the underlying mechanisms in the brain remain to be fully elucidated. Here, we demonstrate that long-term voluntary running lowered ΔFOSB-positive cell numbers in the SCN in both male and female rats, an effect that was more prominent in females than in males and was independent of total running distance. Furthermore, the number of ΔFOSB-positive cells in the SCN fluctuated across the estrous cycle in female sedentary rats, with the highest levels being observed during diestrus and the lowest levels during proestrus. This cyclicity was completely absent in female runners. Estradiol replacement following ovariectomy in sedentary females lowered SCN ΔFOSB-positive cell numbers. Finally, SCN ΔFOSB was predominantly expressed in neurons in the ventral core, but not dorsal shell, region of the SCN and was co-expressed to some degree with VIP, but not AVP.

A consistent finding in rodent studies is that long-term VWR increases ΔFOSB in several brain regions, including nucleus accumbens, dorsomedial striatum, dorsal raphe nucleus, hippocampal subregions, and medial prefrontal cortex [43,44,45,46,47,48,49,50,51,52]. Based on these observations, we hypothesized that VWR would also increase ΔFOSB in the SCN. Contrary to our hypothesis, we observed that voluntary running lowered ΔFOSB-positive cell numbers in the SCN of male and female rats compared to sedentary controls. To the best of our knowledge, one study has previously reported a decrease in FOSB/ΔFOSB following VWR, namely in the basolateral amygdala of individually-housed, running prairie voles compared to individually-housed sedentary prairie voles [51].

Using an inducible transgenic mouse model it was demonstrated that ΔFOSB activates or represses expression of many genes in a time-dependent manner [53]. This also occurs during physiological conditions, as accumulation of ΔFOSB, in striatal neurons following chronic amphetamine exposure or in dentate gyrus neurons following recurrent seizures, represses expression of *c-fos*, another Fos family gene and a classic marker for cellular activation, via epigenetic mechanisms [54,55]. Furthermore, learning-mediated accumulation of hippocampal ΔFOSB inhibits production of cFOS in granule cells of the dentate gyrus, but not in neurons of the CA1, and is involved in memory processes [30]. Finally, viral-mediated overexpression of ΔFOSB decreases excitability of dorsal hippocampal CA1 neurons [56], consistent with the notion that target genes of ΔFOSB include critical regulators of glutamatergic synaptic strength, including certain AMPA-type glutamate receptor subunits (*e.g*. glutamate receptor 2), Ca2+/calmodulin-dependent protein kinase IIα, and cyclin-dependent kinase 5 [20,57,58,59,60,61].

Taken together, these findings suggest that repression of SCN ΔFOSB by VWR might be mirrored by greater neuronal excitability, compared to sedentary controls, and a higher amplitude of neuronal activity rhythms. Potentially, this may sensitize the SCN, and especially the ventral core, to environmental changes in entrainers, like light. In rats living in the wild, an increased excitability of the SCN may facilitate responsiveness and potentially improve synchronization to the environment and its (seasonal) changes. This is in line with the observation that VWR improves entrainment to a new circadian phase of the light-dark cycle in mice [62]. A recent review and consensus statement about travel fatigue and jet lag in athletes concluded that there is a lack of evidence for interventions that reduce the symptoms of jet lag, but exercise was identified as a potential strategy [63]. Our findings support the hypothesis that exercise may be a potential strategy to facilitate adaptation to a new circadian phase and thus reduce jet lag symptoms in humans, although this hypothesis remains to be investigated in humans. Thus, VWR-mediated repression of ΔFOSB, and its subsequent effects on neuronal excitability, may act as a molecular mechanism in the SCN linking physical exercise to altered circadian input and output.

Many rodent studies focusing on drugs of abuse and their addictive effects have revealed an important role for the induction of ΔFOSB in medium spiny neurons of the nucleus accumbens, in particular in relation to dopamine signaling and specifically via the dopamine d1 receptor (DRD1) [20]. For a long time it was thought that *ΔFosB* mRNA is generated constitutively above a certain level of *FosB* mRNA expression [64]. However, it was recently demonstrated that dopamine signaling can synergize with the TGFβ superfamily receptor ALK4 to potentiate the production of ΔFOSB in medium spiny neurons of the nucleus accumbens through activation of the RNA-binding proteins PCBP1 and SMAD3 [65]. Thus, it is not unlikely that dopamine- and/or TGFβ-signaling is also involved in the modulation of SCN ΔFOSB, especially since many DRD1-expressing neurons are present in the SCN [66,67]. Additional studies are necessary to determine if dopamine- and/or TGFβ-signaling are also involved in the VWR-mediated suppression of SCN ΔFOSB and which neuronal markers, aside from VIP, identify the SCN neuron subtypes where this suppression occurs.

Which other molecular mechanisms can modulate SCN ΔFOSB? In this study we first observed that ΔFOSB-positive cell numbers in the SCN fluctuated across the estrous cycle in female sedentary rats, with the highest levels during diestrus and the lowest levels during proestrus. The estrous cycle is characterized by distinct hormonal changes, with an estrogen and progesterone surge during proestrus, and a second, but smaller, progesterone surge during metestrus/diestrus [68,69,70]. Estrogen signaling regulates VWR behavior, as female rats show cyclic variations in running linked to the estrous cycle, with ovariectomy reducing VWR and estrogen replacement restoring VWR [71]. Interestingly, we observed high levels of SCN ΔFOSB during metestrus/diestrus, which seems to coincide with low estrogen levels and a progesterone peak. However, physiological progesterone levels did previously not affect SCN *Per1*-Luc rhythms [72]. Therefore, we hypothesized that either an indirect pathway, or signaling via estrogen receptors and/or other receptors in the SCN, mediated the cyclicity in SCN ΔFOSB. Thus, we used estrogen replacement in an OVX background to mechanistically determine that estradiol replacement following ovariectomy lowers ΔFOSB-positive cell numbers in the SCN, indicating that the fluctuation of ΔFOSB-positive cell numbers during the estrous cycle are mediated, at least in part, by estradiol. Circulating estrogenic hormones, including estradiol, are well known to affect the circadian system in rodents [71,73,74], but it is not clear whether these are direct effects at the level of the SCN or indirect via afferent projections. Whereas no clear observable effects of estradiol treatment on SCN explants were observed [72], nor of the estrous cycle itself on the rhythmicity of the expression of several major clock genes [75], estradiol can directly alter neuronal activity in the SCN [76]. Indeed, estradiol increased firing frequency and excitatory synaptic transmission of neurons in ventromedial SCN explants of male rats, and this effect was abolished by the administration of an estradiol antagonist [77]. Moreover, although ΔFOSB was not assessed directly, cFOS levels in the SCN were reported to be lower following ovariectomy in rats, and estradiol treatment successfully restored SCN cFOS levels during the light phase [78,79,80]. Furthermore, the start of feminizing gender affirming hormone treatment (including estradiol) in transgender humans, is associated with a 20 min forward chronotype shift [81]. Because ΔFOSB inhibits production of cFOS in mouse striatal neurons or dentate gyrus granule cells [30,55], and post-partum estradiol withdrawal increases ΔFOSB in mouse nucleus accumbens [82], ΔFOSB might thus mediate some of the effects of estradiol on SCN function. For example, it was recently demonstrated in the nucleus accumbens that low-estrogenic females respond to acute cocaine by opening neuronal chromatin enriched for the sites of ΔFOSB, whereas high-estrogenic females respond to cocaine by preferential chromatin closing, providing a mechanism for limiting cocaine-driven chromatin and synaptic plasticity [83]. Similar mechanistic interactions between estrogen and ΔFOSB might also modify the response of the SCN to physiological stimuli, which could potentially explain chronotype differences observed between human sexes [84].

SCN ΔFOSB was predominantly localized to the lateral part of the ventral core region, but not to dorsal shell region (Supplemental Figs. 1a,b). Current understanding of the functional differences between these subregions indicates that the SCN core region receives, integrates, and converts various kinds of entraining information, amongst others to the dorsal shell, whereas the SCN shell region generates circadian oscillations and sends these entrained signals to the rest of the brain [42,85]. The ventral core region receives entraining information from several sources, including photic input via the retinohypothalamic tract (RHT), serotonin (5-HT) input from the raphe nuclei, dopaminergic input from the ventral tegmental area, and neuropeptide y (NPY) input from the intergeniculate leaflet (IGL) [86]. As all these areas also express estrogen receptors [87], we also cannot exclude the possibility of an indirect estrogen or progesterone effect. An interesting observation in this regard is a study in golden hamsters by LeSauter & Silver [88] who identified a SCN subregion involved in the regulation of locomotor activity. The localization of this subnucleus, characterized by the expression of the calcium-binding protein Calbindin-D_28K_ (CaBP), shows strong similarity to the ΔFOSB-expressing subnucleus in the lateral part of the core SCN. In earlier SCN transplantation experiments the strength of the restored rhythm correlated with the number of CaBP-positive cells in the graft. Unfortunately, in the rat SCN, CaBP expression does not show such a localized expression but is scattered throughout the SCN [89]. Additional studies are necessary to determine which brain regions and/or signaling cascades are involved in the effects of VWR on SCN ΔFOSB.

A key contribution of the present study involves our demonstration that ΔFOSB-positive cell numbers in the SCN are modulated by voluntary running in both male and female rats and by the estrous cycle via, at least in part, estradiol in female rats. These findings increase our mechanistic understanding of how physical exercise and the estrous cycle regulate ΔFOSB in the SCN and how physical exercise potentially sensitizes the SCN. Targeting the key components of these mechanisms may lead to interventions that can help optimize the circadian impact of physical exercise or counteract the effects of circadian dysregulation.

## Data Accessibility Statement

Data will be made available on reasonable request.

## Additional File

The additional file for this article can be found as follows:

10.5334/jcr.257.s1 Supplemental figures. Supplemental Figures 1 to 3.
