# Supplementary material for: Voluntary Running and Estrous Cycle Modulate ΔFOSB in the Suprachiasmatic Nucleus of the Wistar Rat
Source: J Circadian Rhythms. 2025 May 19;23:7. doi: 10.5334/jcr.257 (PMC12101111; doi:10.5334/jcr.257)
Supplement: Supplemental figures. — Supplemental Figures 1 to 3. [file jcr-23-257-s1.pdf]

Supplemental figures

Supplemental Figure 1

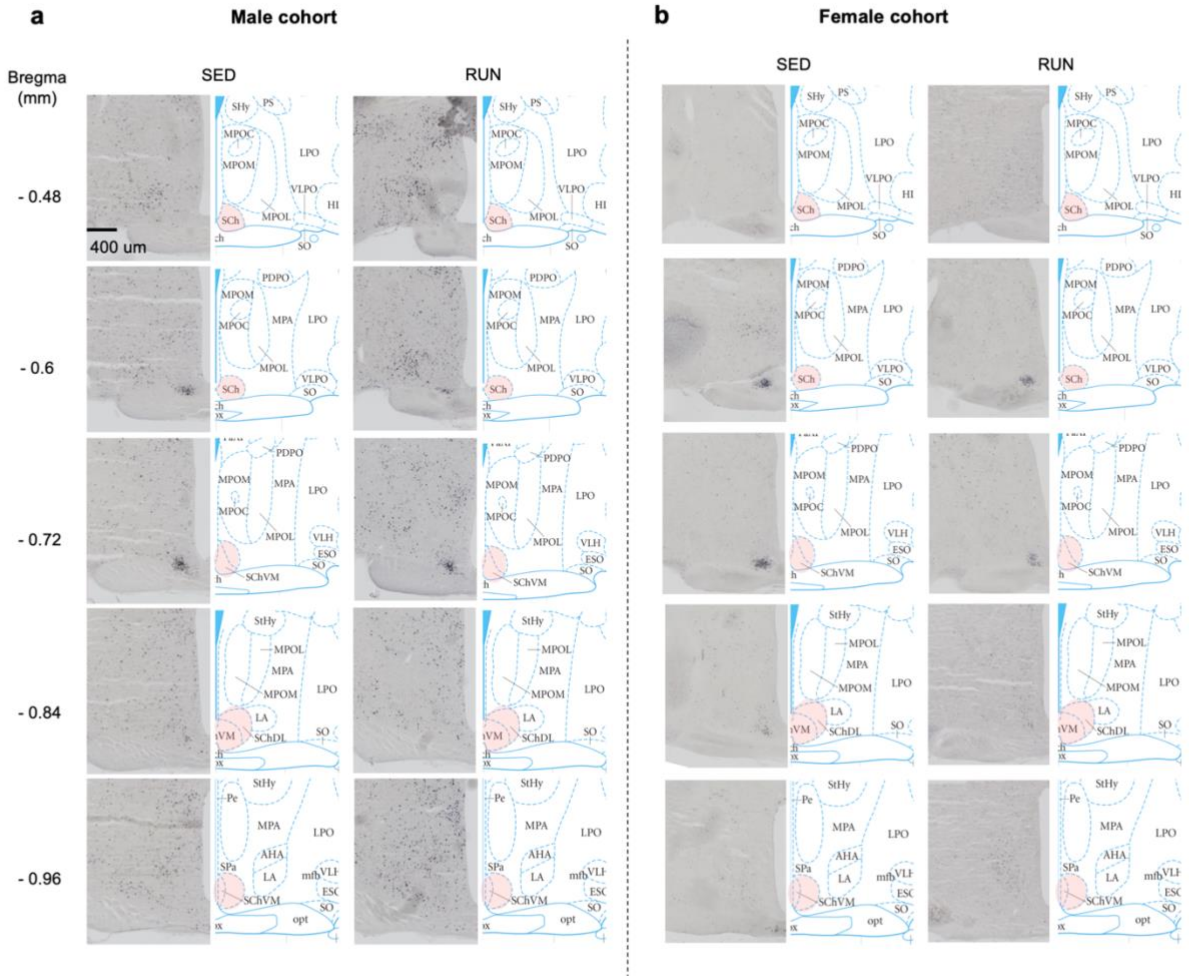

Supplemental figure 1. **SCN ΔFOSB expression in male and female runners and sedentary controls**.
(a,b) Representative microphotographs of SCN brain slices (left side) shown with the respective rat brain atlas plate (right side; 6th edition by George Paxinos and Charles Watson) of a male (a) and a female (b) runner (RUN) and sedentary (SED) control. Pink-shaded areas indicate the SCN region.

Supplemental Figure 2

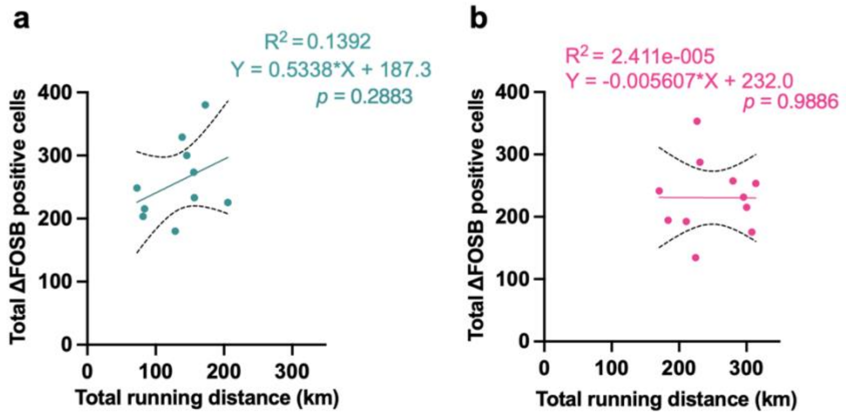

Supplemental figure 2. **Correlations between SCN ΔFOSB and running distance**. Correlation between total running distance and the number of ΔFOSB-positive cells in the SCN (between bregma -0.6mm and -0.84mm) in the primary (a) male and (b) female running cohorts. Solid line represents simple linear regression, black dotted lines represents 95% confidence bands of the best fit.

Supplemental figure 3

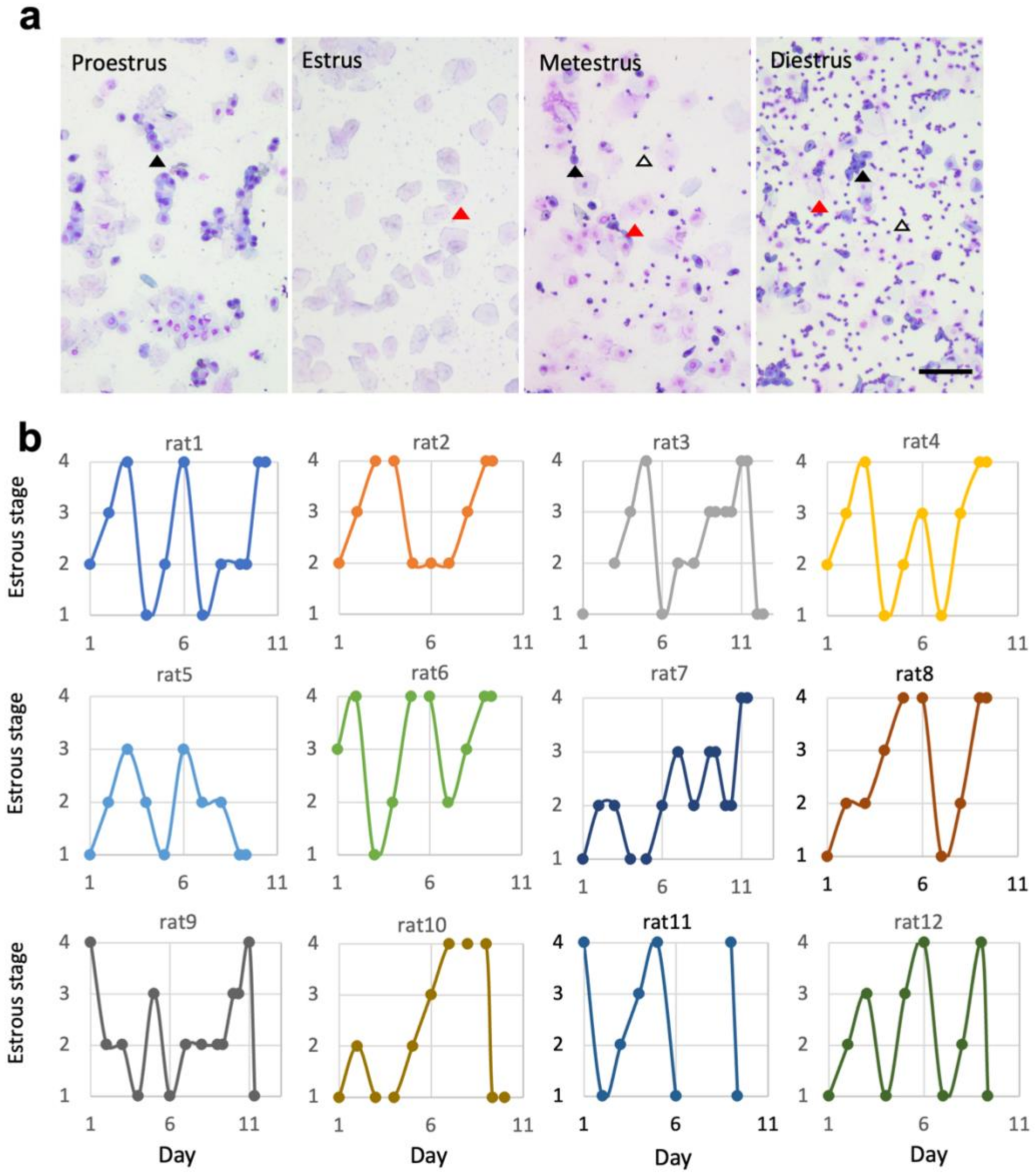

Supplemental Figure 3. **Representative microphotographs of vaginal smear cytology at each stage of rat estrous cycle.** (a) Nucleated epithelial cells (black arrowheads), cornified cells (red arrowheads), and leukocytes (white arrowheads) at different ratios depending on stages of the cycle (scale bar = 100µm). (b) Estrous cycle tracking of 12 individual rats of the independent and sedentary female cohort to replicate the effects of estrous cycle on SCN ΔFOSB (y-axis: 1 = proestrus, 2 = estrus, 3 = metestrus, 4 = diestrus).
